# Investigating Thermotolerance of Thylakoid Processes in Two Cotton Species using Rapid Induction Fluorescence

**DOI:** 10.1002/pei3.70181

**Published:** 2026-06-25

**Authors:** Comfort O. Adegbenro, Ved Parkash, John L. Snider, Viktor Tishchenko

**Affiliations:** ^1^ Department of Crop and Soil Sciences University of Georgia Tifton Georgia USA; ^2^ Department of Crop and Soil Sciences University of Georgia Griffin Georgia USA

**Keywords:** chlorophyll fluorescence, heat stress, pima cotton, thermotolerance, upland cotton

## Abstract

High temperatures can hinder stand establishment, seedling growth, and photosynthetic processes in cotton. Yet, interpretations of thermotolerance in thylakoid processes often depend on whether measurements follow chronic or acute heat exposure, and species‐level differences in these responses remain poorly characterized. This study evaluated the effects of chronic high temperatures (40°C/30°C) on key thylakoid processes in four‐week‐old Upland and Pima cotton seedlings and assessed their acclimation potential using rapid induction chlorophyll fluorescence across a range of incubation temperatures. As these processes were inferred from OJIP parameters rather than direct measurements of photosystem activity, they are interpreted as fluorescence‐based indicators of thylakoid function. Chronic heat enhanced photosystem I (PSI)‐related parameters in both species, with Upland exhibiting larger increases (44%–46%) compared to Pima (39%–40%), whereas photosystem II (PSII) photochemistry remained largely stable under chronic heat, declining by less than 2% at 40°C and by 6%–8% at 45°C under acute exposure. Acute temperature responses closely mirrored chronic patterns, and Upland showed higher thermal optima for PSI quantum yield and overall performance (40°C) than Pima (35°C), suggesting greater PSI electron‐sink capacity. Collectively, these fluorescence‐derived results suggest that PSI‐related processes distinguish species‐level thermotolerance in cotton. Upland's stronger enhancement of PSI acceptor‐side capacity under both sustained and transient heat exposure points to a more robust acclimation strategy, whereas Pima shows limited PSI adjustment despite maintaining PSII efficiency. These findings clarify how thylakoid processes respond to heat across timescales and may help guide the development of heat‐resilient cotton as growing‐season temperatures rise.

## 1 Introduction

Cotton (*Gossypium spp*) is globally significant, especially in the apparel, animal feed, and food industries (Liu et al. 2012; OECD/FAO 2023). Cotton production contributes to the economic growth of many countries, including the United States (USDA‐ERS 2022). High seedling vigor is important in cotton production to ensure stand establishment and reduce susceptibility to early‐season pests (Snider and Oosterhuis 2015; Raphael et al. 2017; Hand et al. 2021; Liu et al. 2015). Hence, temperature extremes or other stressors considered unsuitable for key processes and growth stages may significantly penalize stand establishment and increase the risk of crop loss (Snider et al. 2021). While low temperatures during the early spring months are the most common limitation to seedling growth, potentially growth‐limiting high temperatures are not uncommon in the weeks that follow planting in the southeastern US (Virk et al. 2021).

It is well established that reproductive development in cotton is sensitive to high‐temperature extremes (Reddy, Hodges, and Reddy 1992; Reddy et al. 1997; Snider and Oosterhuis 2011; Snider et al. 2009; Luqman et al. 2025); however, detrimental effects of heat stress on early‐season growth and photosynthetic processes have also been observed (Reddy et al. 2017; Virk et al. 2021; Raphael et al. 2017; Kaur, Snider, Parkash, et al. 2023; Parkash et al. 2024). High temperatures above 35°C limited leaf area development, height, and total shoot dry weight of cotton seedlings (Virk et al. 2021), whereas in other studies, temperatures above 30°C were sufficient to cause significant declines in biomass accumulation (Reddy et al. 1991; Kaur, Snider, Parkash, et al. 2023). Although genotypic differences in seedling growth response to high temperature have been observed for cotton, a 40°C/30°C temperature regime consistently caused reductions in leaf area and total dry weight, irrespective of cultivar (Kaur, Snider, Parkash, et al. 2023). Similarly, declines in photosynthesis under acute high temperature stress (35°C to 40°C) have been well documented (Crafts‐Brandner and Salvucci 2000; Law and Crafts‐Brandner 1999). Heat‐induced limitations to photosynthesis have been attributed to different factors, ranging from limitations to electron transport and RuBP regeneration to the inactivity of rubisco (Wise et al. 2004; Scafaro et al. 2023; Schrader et al. 2004; Crafts‐Brandner and Law 2000), and potentially even declines in photosynthetic pigments (Thompson et al. 2022; Snider et al. 2009; Snider, Oosterhuis, and Kawakami 2010; Parkash et al. 2024). High temperatures also promote reactive oxygen species accumulation, and cotton genotypes differ in their capacity to maintain oxidative homeostasis and overall thermotolerance (Luqman et al. 2025; Khan et al. 2026).

Chlorophyll fluorescence has been widely utilized to identify functional limitations to photosynthesis under a wide range of conditions (Maxwell and Johnson 2000). Measurements that characterize the polyphasic rise in chlorophyll fluorescence of dark‐adapted leaves are often termed “OJIP” assays after the different steps measured in the fluorescence transient (Strasser et al. 2000; Strasser et al. 2010). Specifically, parameters measured under dark‐adapted conditions using OJIP fluorescence (Strasser et al. 2000) include structural indicators, performance indices, and quantum efficiencies of the photosynthetic apparatus under abiotic stress (Kaur, Snider, Paterson, et al. 2023; Virk et al. 2021; Snider et al. 2018; Strasser et al. 2010; Strauss et al. 2006). For cotton plants subjected to long‐term heat stress, PSII antenna size and reaction center‐specific fluxes (ABS/RC, TR_0_/RC, and DI_0_/RC) did not differ from plants grown under optimal temperature regimes (Virk et al. 2021). In contrast, the relative abundance of PSI with respect to PSII (Δ_VIP_) increased by 8% when the temperature increased from 30°C to 35°C and 40°C. This is in contrast with declines in Δ_VIP_ observed in the literature for other forms of abiotic stress (Ceppi et al. 2012; Oukarroum et al. 2009). Performance indices and quantum efficiencies of various steps in the thylakoid reactions were influenced by chronic heat stress. The fluorescence index representing the contribution of the light‐dependent reactions to primary photochemistry (F_v_/F_0_) and the photosynthetic performance index (PI_ABS_) declined by 13% and 22%, respectively, when growth temperature increased from 35°C to 40°C (Virk et al. 2021). In the same study, the quantum efficiency of energy trapping (Φ_Po_), and intersystem electron transport (Φ_Eo_) declined at 40°C by 4% and 12%, respectively. In comparison, the total performance index (PI_TOTAL_) and the quantum efficiency of PSI electron acceptor reduction (Φ_Ro_) were not influenced by temperature increases up to 40°C (Virk et al. 2021).

Importantly, heat tolerance can differ drastically depending on the thylakoid process evaluated (Virk et al. 2021; Law and Crafts‐Brandner 1999; Kaur, Snider, Parkash, et al. 2023; Parkash et al. 2024; Snider et al. 2015). For example, the maximum quantum yield of energy trapping by photosystem II (F_v_/F_m_ or ϕ_P0_) has been generally considered stable under heat stress well above 40°C (Law and Crafts‐Brandner 1999; Kaur, Snider, Parkash, et al. 2023; Snider, Oosterhuis, and Kawakami 2010; Snider et al. 2013), while other quantum efficiencies, structural indicators, and performance indices exhibit greater heat sensitivity (Virk et al. 2021; Hu et al. 2018). However, interpretations of high‐temperature effects and thermotolerance of thylakoid processes could depend on whether measurements are done following chronic or acute high‐temperature exposure (Bibi et al. 2008; Sarwar et al. 2023; Kaur, Snider, Paterson, et al. 2023; Kaur, Snider, Parkash, et al. 2023; Virk et al. 2021; Sethar et al. 2002). This is because the growth environment could alter the thermotolerance of thylakoid processes through acclimation (Kaur, Snider, Parkash, et al. 2023; Kaur, Snider, Paterson, et al. 2023; Snider et al. 2013).

Rapid temperature response curves have been employed to estimate the thermotolerance of thylakoid processes by exposing dark‐adapted leaves to increasing incubation temperatures and calculating temperature thresholds causing a 15% decline (T_15_) in each process (Chastain et al. 2016; Froux et al. 2004; Kaur, Snider, Parkash, et al. 2023; Hu et al. 2018; Snider et al. 2015). For instance, Snider et al. (2015) noted that the lowest T_15_ value for Φ_Pο_ was 45.2°C during a relatively mild growing season. However, corresponding T_15_ values for variable fluorescence (F_v_/F_0_), the quantum yield of intersystem electron transport (Φ_Eo_), and the quantum yield of photosystem I electron acceptor reduction (RE_ο_/ABS or Φ_Rο_) were 34.1°C, 37.6°C, and 42.0°C, respectively. Similarly, using the same approach, Hu et al. (2018) showed that the quantum efficiency of energy trapping by PSII and electron acceptor reduction by PSI (Φ_Ro_ and Φ_Po_) were more heat‐tolerant than the quantum efficiency of inter‐photosystem electron transport (Φ_Eo_) or photosynthetic performance indices that were dependent on electron transport (PI_TOTAL_ and PI_ABS_). The authors also documented upward shifts in T_15_ across nearly every process in plants grown under optimal day/night temperatures (30°C/20°C) compared with those grown under suboptimal temperatures (20°C/15°C).

Cotton cultivars have been shown to differ in heat tolerance of thylakoid processes (Kaur, Snider, Parkash, et al. 2023; Hejnák et al. 2015; Kaur, Snider, Paterson, et al. 2023; Virk et al. 2021). Furthermore, as thylakoid processes can exhibit heat acclimation (Kaur, Snider, Parkash, et al. 2023; Froux et al. 2004), cotton cultivars have shown significant differences in seedling heat tolerance and acclimation potential of PSII (Kaur, Snider, Paterson, et al. 2023; Kaur, Snider, Parkash, et al. 2023; Virk et al. 2021; Adegbenro et al. 2025). Another study comparing photosynthetic thermotolerance and growth responses of Upland ( *Gossypium hirsutum* ) and Pima ( *Gossypium barbadense* ) cultivars showed that the Pima cultivar was the most heat‐sensitive for early‐season growth and Φ_Po_, whereas it was more heat‐tolerant for Φ_Eo_ and Φ_Ro_ than other tested Upland cultivars (Kaur, Snider, Parkash, et al. 2023). Despite the strength of this study, photosynthetic thermotolerance was addressed for only three thylakoid responses (Φ_Po_, Φ_Eo_ and Φ_Ro_) using rapid temperature response curves (Kaur, Snider, Parkash, et al. 2023), and no attempt was made to distinguish between chronic and acute heat stress effects on OJIP‐derived parameters. Upland and Pima cotton are the first and second most widely grown species of cotton in the world (Unruh and Silvertooth 1996; Holladay et al. 2021). Within the US, Pima is primarily grown in the hot, arid southwest, whereas Upland is grown across a much broader range of environments that encompasses the entire southern half of the country (Unruh and Silvertooth 1996). As a result, it was hypothesized in the current study that (1) the two species would exhibit significant differences in thylakoid responses to chronically high growth temperature and (2) these species‐specific differences in thylakoid responses would be explained by differences in the acclimation potential of the two species. Hence, the objectives of this study were: (1) to assess the effect of chronic high temperature exposure on key thylakoid processes in seedlings of two cotton species, and (2) to assess the acclimation potential of thylakoid processes using rapid temperature response curves.

## 2 Materials and Methods

### 2.1 Growth Conditions and Treatments

This study was conducted in two walk‐in growth chambers at the Georgia Envirotron facility, University of Georgia, Griffin, GA. Two different temperature regimes were used: (1) 30°C during the day and 20°C at night (optimal temperature) and (2) 40°C during the day and 30°C at night (high temperature) (Kaur, Snider, Parkash, et al. 2023; Virk et al. 2021; Burke and Wanjura 2010). Two cotton species were studied: (1) Upland ( *G. hirsutum* ) and (2) Pima ( *G. barbadense* ) (Table 1). The Upland cultivar Deltapine 1646 B2XF and the Pima cultivar Deltapine 341 RF were used, and both were among the top commercially available upland and Pima cultivars commercially produced in the United States at the time of this experiment (Hutmacher 2021; USDA‐AMS 2020; Hand et al. 2023).

**TABLE 1:** Experimental design and treatment structure for each cotton species, showing growth‐temperature treatments, plant developmental stage at the time of measurement, and the leaf position measured at each point of chlorophyll fluorescence measurement.

Species	Growth temperature (°C)	Plant age/stage at measurement	Leaf position measured
Upland (Deltapine 1646 B2XF)	30	4 weeks after planting (vegetative stage)	Uppermost fully expanded, mainstem leaves
	40	4 weeks after planting (vegetative stage)	Uppermost fully expanded, mainstem leaves
Pima (Deltapine 341 RF)	30	4 weeks after planting (vegetative stage)	Uppermost fully expanded, mainstem leaves
	40	4 weeks after planting (vegetative stage)	Uppermost fully expanded, mainstem leaves

All environmental conditions, except for temperature, were kept consistent across both growth chambers. Photosynthetically active radiation was maintained at an intensity of 800 μmol m^−2^ s^−1^ during the day, with a photoperiod of 14 h of light and 10 h of darkness. This is a similar photoperiod to what is normally experienced by the cotton crop in the early season in Georgia. The plants were grown under the specified temperature regimes for four weeks following planting, before floral initiation. This study was conducted in two separate experimental runs. To minimize potential chamber effects, growth‐temperature regimes were reassigned to different growth chambers between the two experimental runs, so that no regime remained in the same chamber across runs. In each experimental run, there were 10 pots of each cotton species randomized in each growth chamber to fulfill various study objectives. Specifically, five pots were used for survey measures of dark‐adapted, predawn chlorophyll fluorescence, and five pots were used for fluorescence‐incubation temperature experiments.

Plants were grown in 8‐l pots filled with a nursery growing media (PRO‐MIX BX, Premier Tech Growers, and Consumers, Quakertown, PA). Three seeds were planted per pot, and after seedling emergence, two plants were removed to maintain one healthy plant per pot. All plants were well‐watered by irrigating to full pot capacity daily throughout the experiment, ensuring that water availability did not limit growth. Starting one week after planting, the plants were fertilized twice weekly with a water‐soluble complete fertilizer (P.F.I. 20–20‐20 Multi‐Purpose Water Soluble Fertilizer, Plant Foods). Specifically, each pot was fertilized with 50 mL of a 0.8% w/v solution of the water‐soluble fertilizer.

### 2.2 Chlorophyll Fluorescence Assessments at Growth Temperature

At the end of the four‐week period, chlorophyll fluorescence measurements were conducted on five plants from each cotton species in each growth chamber during both experimental runs. These measurements were performed on uppermost, fully‐expanded, mainstem leaves using a portable photosynthesis system equipped with an integrated leaf chamber fluorometer (LI‐6800, Li‐COR Biosciences, Lincoln, NE, USA). Dark‐adapted chlorophyll fluorescence measurements were taken during pre‐dawn hours (between 0400 and 0600 h). This was done to ensure that plants were dark‐adapted for as long as is realistically possible in a natural setting. However, the temperature of the growth chamber was then adjusted to the daytime setting, and the plants were incubated for 20 min prior to obtaining dark‐adapted measurements. This approach ensured that measurements were done at the highest temperature for both treatments. For the dark‐adapted assessments, all environmental conditions within the leaf chamber were kept consistent across treatments, except for air temperature. Specifically, the photosynthetically active radiation (PAR) intensity was maintained at 0 μmol m^2^s^−1^, the reference CO_2_ concentration was set at 400 μmol mol^−1^, relative humidity was maintained at 60% ± 10%, and the air temperature of the leaf chamber was adjusted according to the growth temperature treatment.

Chlorophyll fluorescence was assessed over a 6 cm^2^ leaf area using an induction flash to generate chlorophyll transient curves. With the exposure of the photosynthetic sample to a saturating flash of light, fluorescence intensity changes from the intensity observed in the ground state (F_0_) to maximum fluorescence intensity (F_m_). From this induction curve, intensities of fluorescence at the start of the measurement (O step; F_0_), inflection point (J step; 2 milliseconds), intermediary peak (I step; 20 milliseconds), peak (P step; F_m_), and initial slope of the curve are recorded (Strasser and Govindjee 1992). Based on the theory of energy fluxes in biomembranes, it is said that the different steps and phases of induction curves are associated with the redox states of photosystem II, the efficiency of electron transfer beyond Q_A_, and electron transfer to electron acceptors by photosystem I (Strasser et al. 2010). From the recorded fluorescence intensities at O, J, I, and P steps and the initial slope, a number of parameters describing the structural and functional state of the photosynthetic apparatus were derived using formulas provided in the available literature (Strasser et al. 2010; Strauss et al. 2006). Structural and energy flux‐related parameters: (1) RC/CS_0_: density of reaction centers (RC) of PSII within the excited cross section (CS_0_), (2) ABS/RC: absorption flux per RC (effective antenna size of active reaction centers of PSII), (3) TR_0_/RC: energy flux contributing to reduction of Q_A_ per RC, (4) ET_0_/RC: electron flux driving reduction of electron acceptors located past Q_A_ in the electron transport chain, (5) RE_o_/RC: electron flux driving reduction of electron acceptors by PSI, (6) DI_0_/RC: dissipation energy flux per RC, and (7) Δ_VIP_: indicator of relative abundance of PSI with respect to PSII. Quantum efficiency related parameters: (1) Φ_Po_: maximum quantum yield of primary photochemistry of PSII of a dark‐adapted leaf, (2) Φ_Eo_: quantum yield of electron transport to electron acceptors beyond Q_A_, and (3) Φ_Ro_: quantum yield of electron transport to electron acceptors at the PSI acceptor side.

Performance indices included: (1) PI_ABS_: this performance index represents the energy conservation from photons absorbed by the PSII antenna to the reduction of electron acceptors present beyond Q_A_ in the electron transport chain; (2) PI_TOTAL_: this performance index represents energy conservation from photons absorbed by PSII antenna to the reduction of electron acceptors at the PSI acceptor side, and (3) F_v_/F_0_: this represents the contribution of light‐dependent reactions to primary photochemistry (Strasser et al. 2010; Strauss et al. 2006).

The complete datasets showing chlorophyll fluorescence responses to growth temperature and cotton species are provided as Data S1 in the supporting information, with variable definitions described in the accompanying README (see File S1).

### 2.3 Response of Chlorophyll Fluorescence Parameters to Incubation Temperature

To generate rapid temperature response curves for each experimental unit, at the conclusion of the four‐week period, the response of dark‐adapted chlorophyll fluorescence to increasing incubation temperature (30°C, 35°C, 40°C, and 45°C) was assessed. Five plants from each species from each growth temperature were transferred to a growth chamber maintained at 30°C. All other environmental conditions in this chamber were kept consistent with those in the growth chambers used for the temperature treatments. The fluorometer settings and environmental conditions within the LI‐6800 leaf chamber were aligned with those used for the previous dark‐adapted chlorophyll fluorescence measurements. The air temperature of the leaf chamber was adjusted to match that of the growth chamber during the measurements. Following initial measurements at 30°C, the temperature of the growth chamber and the leaf chamber on the LI‐6800 were increased to the next target incubation temperature. Approximately 15 min was required for chamber temperature to reach the target temperature, and once the target temperature was reached, plants were incubated for another 5 min at each temperature prior to conducting OJIP fluorescence measurements. Because measurements were made with the LI‐6800 rather than a conventional fast‐fluorescence instrument, gas‐exchange and fluorescence data were logged only once net photosynthetic rate had reached steady state (~2 min). Fluorescence data at each temperature were logged only after leaf temperature and net photosynthesis had stabilized, confirming physiological equilibration prior to measurement. Furthermore, short incubation periods of this kind (5 min) are consistent with established fluorescence‐temperature response protocols for cotton and other species, in which incubation times of less than or equal to 6 min yield stable temperature‐response curves (Epron 1997; Ladjal et al. 2000; Kaur, Snider, Paterson, et al. 2023; Snider, Oosterhuis, and Kawakami 2010; Snider, Choinski, and Slaton 2010; Hu et al. 2018). The parameters recorded at each incubation temperature included fluorescence intensities at the O, J, I, and P steps, as well as the initial slope of the induction curve, as described above. Φ_Po_, Φ_Eo_, Φ_Ro_, F_v_/F_0_, PI_ABS_, and PI_TOTAL_ at each incubation temperature were calculated from the recorded data. All measurements were performed on uppermost, fully‐expanded, mainstem leaves (fourth node below the terminal) at each incubation temperature.

The complete incubation temperature response datasets are provided as Data S2 in the supporting information. Variables are described in the accompanying README document (see File S1).

### 2.4 Statistical Analysis

All statistical analyses were performed using JMP Pro 18 (SAS Institute Inc., Cary, NC, USA), and figures were prepared using SigmaPlot 16.0 (Systat Software Inc.).

#### 2.4.1 Objective 1

The study was conducted over two experimental runs, with each run treated as a block. During both runs, growth temperature treatments were randomly assigned to different growth chambers. Within each chamber, two cotton species were nested, with five pots allocated for each species. Each pot was considered a replicate, resulting in ten replications for each cotton species at each growth temperature across both experimental runs. To assess the effects of growth temperature, cotton species, and their interactions on the parameters of interest, a mixed‐effects analysis of variance (ANOVA) was performed. In this analysis, growth temperature (GT), cotton species (CS), and the interaction between GT and CS were treated as fixed effects. Experimental run × replication and experimental run × replication × growth temperature were considered random effects. Treatment means were separated using Fisher's protected LSD test at a significance level of 0.05. Significant interaction effects between growth temperature and cotton species were observed for most parameters where any significant effect was observed; thus, the results of these interactions are the primary focus of this report.

#### 2.4.2 Objective 2

To evaluate the effects of growth temperature (GT), cotton species (CS), incubation temperature (IT), and their interactions, a mixed‐effects ANOVA with repeated measures was employed. In this model, GT, CS, IT, GT × CS, GT × IT, CS × IT, and GT × CS × IT were considered fixed effects. Replication within experimental run × growth temperature × cotton species was treated as a subject, while incubation temperature served as a within‐subjects factor. Experimental run × replication and growth temperature × experimental run × replication were treated as random effects. For post hoc analysis, Fisher's protected LSD test was applied at a significance level of 0.05.

## 3 Results

### 3.1 Temperature and Cotton Species Effects on Structural Indicators

The density of reaction centers (RC) of PSII within the excited cross section (CS_0_) of a leaf (RC/CS_0_), absorbed energy flux per RC (ABS/RC), energy flux contributing to the reduction of Q_A_ per RC (TR_0_/RC), and dissipation energy flux per RC (DI_0_/RC) were not affected by the growth temperature, cotton species, or their interactions (Table 2; full statistical output is provided in Table S1). In contrast, electron flux to electron acceptors beyond Q_A_ per RC (ET_o_/RC) was significantly impacted by the growth temperature only (Table 2). ET_o_/RC was positively affected by high growth temperature, where plants grown in 40°C/30°C (1.38) increased specific flux by 11% relative to plants grown in 30°C/20°C (1.24). Electron flux per RC contributing to reductions of PSI end electron acceptors (RE_o_/RC) was significantly affected by growth temperature, cotton species, and their interaction (Table 2). RE_o_/RC increased from 0.59 in 30°C/20°C to 0.92 in 40°C/30°C for Upland and from 0.58 in 30°C/20°C to 0.81 in 40°C/30°C for Pima. RE_o_/RC was statistically comparable between the two species at 30°C/20°C, whereas at 40°C/30°C, Upland had a significantly higher RE_o_/RC than Pima. Similarly, the relative abundance of PSI with respect to PSII (Δ_VIP_) was significantly affected by growth temperature, cotton species, and their interaction. High temperature exerted a positive impact on Δ_VIP_, and it increased from 0.27 in the 30°C/20°C regime to 0.39 in the 40°C/30°C regime for Upland and from 0.26 at 30°C/20°C to 0.36 at 40°C/30°C for Pima. Regarding species comparisons, Upland exhibited higher Δ_VIP_ than Pima only under high‐temperature conditions.

**TABLE 2:** Effect of growth temperature and cotton species on the density of PSII reaction centers (RC/CS_o_), absorbed energy flux per RC (ABS/RC), flux of excitation energy trapped by a reaction center (TR_o_/RC), electron flux per RC to electron acceptors beyond Q_A_ (ET_o_/RC), electron flux per RC to PSI end electron acceptors (RE_o_/RC), dissipation energy flux per RC (DI_o_/RC), indicator of the size of pool of the final electron acceptors of PSI (Δ_VIP_), maximum quantum yield of primary photochemistry (Φ_Po_), quantum yield of inter‐photosystem electron transfer (Φ_Eo_), and quantum yield of reduction of PSI end electron acceptors (Φ_Ro_), performance index representing contribution of light reactions to primary photochemistry (F_v_/F_0_), photosystem II performance index based on light absorption (PI_ABS_), and performance index representing energy conservation from the absorbed photons to a reduction of PSI end acceptors (PI_TOTAL_).

Category	Parameter	Growth temperature			
		30°C/20°C		40°C/30°C	
		Cotton species		Cotton species	
		Pima	Upland	Pima	Upland
Structural indicators	RC/CS_o_	191 a	196 a	190 a	190 a
	ABS/RC	2.73 a	2.71 a	2.79 a	2.85 a
	TR_o_/RC	2.23 a	2.21 a	2.26 a	2.33 a
	DI_o_/RC	0.5 a	0.5 a	0.54 a	0.53 a
	ET_o_/RC	1.28 b	1.19 b	1.36 a	1.39 a
	RE_o_/RC	0.58 c	0.59 c	0.81 b	0.92 a
	Δ_VIP_	0.26 c	0.27 c	0.36 b	0.39 a
Quantum efficiencies	Φ_Po_	0.82 a	0.82 a	0.81 a	0.82 a
	Φ_Eo_	0.47 a	0.44 b	0.49 a	0.49 a
	Φ_Ro_	0.21 c	0.22 c	0.29 b	0.32 a
Performance indices	F_v_/F_0_	4.5 a	4.44 a	4.21 b	4.41 a
	PI_ABS_	2.27 a	1.95 a	2.32 a	2.32 a
	PI_TOTAL_	1.92 c	1.96 c	3.44 b	4.5 a

*Note:* Data shown represent means (*n* = 10; 2 runs × 5 replications). Within a row, mean values not connected by the same letter are significantly different according to Fisher's protected LSD test at a significance level of 0.05.

### 3.2 Temperature and Cotton Species Effects on Quantum Efficiencies

The maximum quantum yield of photosystem II (Φ_Po_) was not affected by growth temperature, cotton species, or their interaction (Table 2). The quantum yield of electron transport to electron acceptors beyond Q_A_ (Φ_Eo_) and the quantum yield of electron transport to electron acceptors at the PSI acceptor side (Φ_Ro_) were significantly responsive to growth temperature, cotton species, and their interaction. At 40°C/30°C, Φ_Eo_ increased relative to the optimum temperature (30°C/20°C), but this response was observed only for Upland, increasing by 11%. Regarding species comparison, at 30°C/20°C, Pima (0.47) had higher Φ_Eo_ than Upland (0.44), while at 40°C/30°C, Φ_Eo_ was comparable between the two species. High temperature had a positive effect on Φ_Ro_ in both species, but the effect was greater in Upland cotton than in Pima. In Upland, Φ_Ro_ increased by 45% in 40°C/30°C relative to 30°C/20°C, while in Pima, it increased by 38%.

### 3.3 Temperature and Cotton Species Effects on Performance Indices

Photosynthetic performance index representing the energy conservation from photons absorbed by PSII antenna to reduction of electron acceptors beyond Q_A_ (PI_ABS_) was not significantly affected by the growth temperature, cotton species, or their interaction (Table 2). In contrast, there was a significant interaction between growth temperature and cotton species for the performance index representing energy conservation from photon absorption by PSII antenna to the reduction of electron acceptors at the PSI acceptor side (PI_TOTAL_; Table 2). PI_TOTAL_ increased in response to high temperature, and it increased from 1.96 in 30°C/20°C to 4.5 in 40°C/30°C for Upland cotton and from 1.92 in 30°C/20°C to 3.44 in 40°C/30°C for Pima. PI_TOTAL_ was comparable between the two species at 30°C/20°C, whereas at 40°C/30°C, Upland maintained a significantly higher PI_TOTAL_ than Pima. The contribution of the light‐dependent reactions to primary photochemistry (F_v_/F_0_) was significantly affected only by the interaction between growth temperature and cotton species. In response to high temperatures, F_v_/F_0_ declined, but this response was observed only in Pima, with a 6.4% decline relative to the optimum temperature. Furthermore, at 40°C/30°C, Upland (4.41) had a higher F_v_/F_0_ than Pima (4.21), while at 30°C/20°C, F_v_/F_0_ was comparable between the two species.

### 3.4 Effect of Cotton Species on Incubation Temperature Responses

**FIGURE 1:**
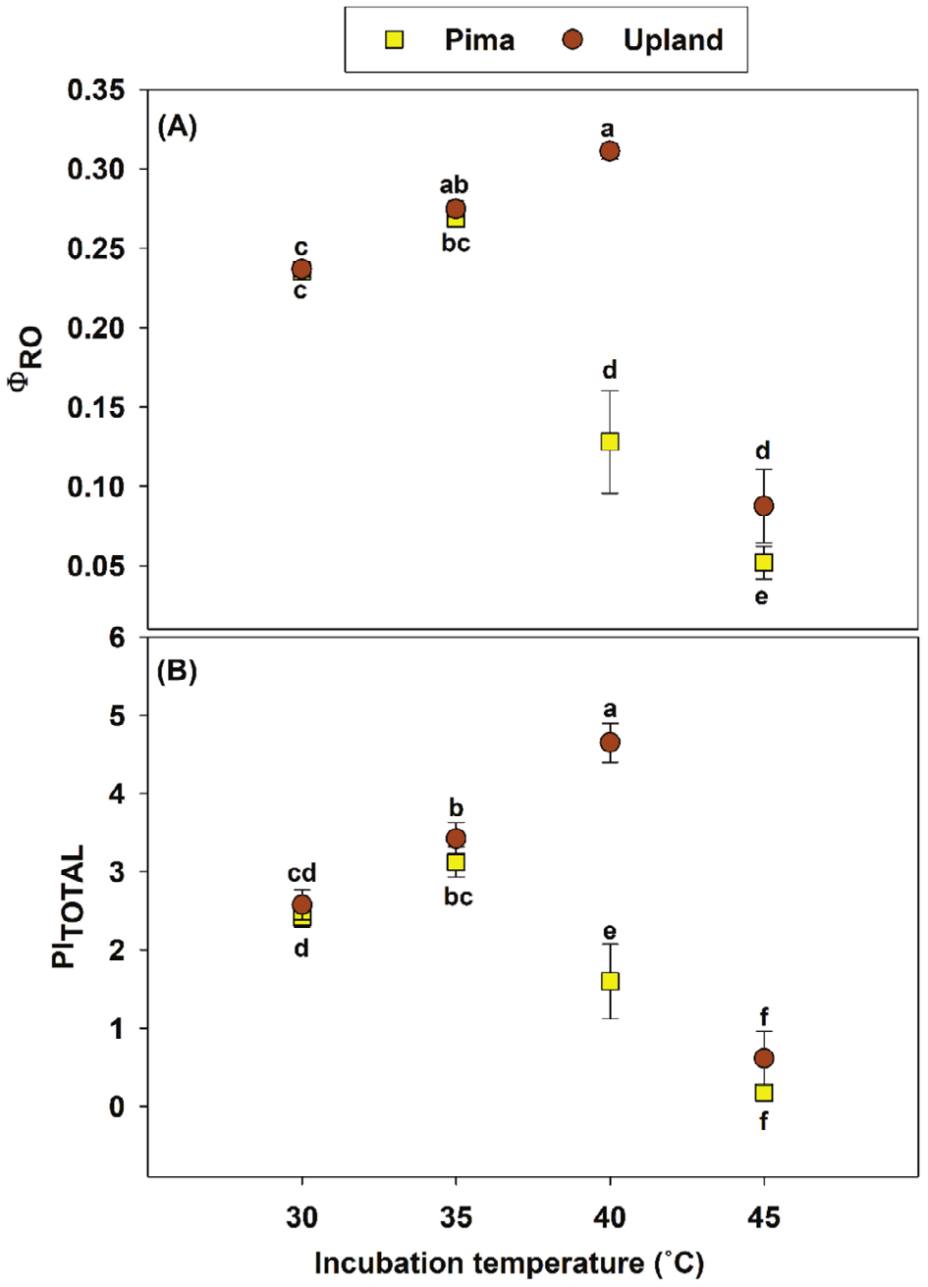
Effect of incubation temperature (30°C, 35°C, 40°C, and 45°C) and cotton species (Pima and Upland) on the quantum yield of reduction of PSI end electron acceptors (Φ_Ro_; A), performance index representing energy conservation from the absorbed photons to a reduction of PSI end acceptors (PI_TOTAL_; B) across two growth temperature regimes (30°C/20°C and 40°C/30°C). Data shown as scatter points represent standard errors; SE (*n* = 20; 2 runs × 5 replications × 2 growth temperature regimes). Values not sharing a common letter significantly differ (*p* < 0.05).

To better understand the effects of growth temperature and cotton species on the sensitivity of specific thylakoid processes to acute high temperature exposure, only significant interactions with incubation temperature are reported. There were no significant three‐way interactions among incubation temperature, growth temperature, and cotton species for any measured variables. However, there was a significant interaction between genotype and incubation temperature only for Φ_Ro_ and for PI_TOTAL_ (Table 3). Therefore, species‐specific responses to incubation temperature are provided for these two variables only (Figure 1).

**TABLE 3:** ANOVA results for incubation temperature (IT), growth temperature (GT), and cotton species (CS) effects on the performance index representing the contribution of light‐dependent reactions to primary photochemistry (F_v_/F_0_), maximum quantum yield of primary photochemistry (Φ_Po_), quantum yield of inter‐photosystem electron transfer (Φ_Eo_), quantum yield of reduction of PSI end electron acceptors (Φ_Ro_), photosystem II performance index based on light absorption (PI_ABS_), and performance index representing energy conservation from the absorbed photons to a reduction of PSI end acceptors (PI_TOTAL_).

Parameters	*p*						
	GT	CS	GT × CS	IT	GT × IT	CS × IT	GT × CS × IT
**F**_**v**_ **/F**_**0**_	< **0.0001**	0.8856	**0.0083**	< **0.0001**	**0.0138**	0.4931	0.8514
**Φ**_**Po**_	< **0.0001**	0.8275	**0.011**	< **0.0001**	< **0.0001**	0.7409	0.6528
**Φ**_**Eo**_	**0.0002**	**0.0266**	< **0.0001**	< **0.0001**	**0.0004**	0.0789	0.0965
**Φ**_**Ro**_	0.2174	< **0.0001**	0.886	< **0.0001**	0.1342	< **0.0001**	0.1799
**PI**_**ABS**_	< **0.0001**	0.9929	< **0.0001**	< **0.0001**	< **0.0001**	0.0703	0.1241
**PI**_**TOTAL**_	**0.0401**	< **0.0001**	0.3144	< **0.0001**	0.2455	< **0.0001**	0.0744

*Note:* Bold values indicate statistical significance at *p* < 0.05.

Φ_Ro_ increased as incubation temperature rose from 30°C to 35°C for both Upland and Pima cotton, with no significant genotypic differences between the two cotton species at either of these temperatures. Φ_Ro_ for Upland and Pima at 35°C increased by approximately 16% and 14%, respectively, relative to 30°C. However, at 40°C, Φ_Ro_ for Upland cotton reached its highest value, increasing significantly by 31%, while Φ_R_o for Pima decreased by 46% compared to 30°C. At the highest incubation temperature (45°C), the lowest Φ_Ro_ values were recorded for both cotton species (Upland = 0.088; Pima = 0.0521), with a significant genotypic difference. Upland cotton at 45°C had a slightly higher Φ_Ro_ value than Pima (Figure 1A). A similar temperature response was observed for PI_TOTAL_, with increases of 29% and 33% for Pima and Upland, respectively, as incubation temperature increased from 30°C to 35°C (Figure 1B). Furthermore, Upland cotton had its highest PI_TOTAL_ value at 40°C, increasing by 81% relative to 30°C, whereas PI_TOTAL_ for Pima declined significantly by 34% at the same incubation temperature. PI_TOTAL_ for both cotton species was lowest (Upland = 0.612; Pima = 0.179) at 45°C and was not significantly different from each other.

Therefore, 40°C was the thermal optimum (the incubation temperature yielding the maximum value) for both processes in Upland cotton, yet 35°C was the optimum temperature in Pima cotton. Since there was no interaction between growth temperature and incubation temperature for either process, prior growth temperature did not alter acute high‐temperature responses in either species.

### 3.5 Effect of Prior Growth Temperature on Incubation Temperature Responses

Significant interactive effects of incubation temperature and growth temperature were observed for Φ_Po_, Φ_Eo_, F_v_/F_0_, and PI_ABS_ (Table 3). No interactions between cotton species and incubation temperature were observed for these measured parameters. This indicates that prior growth temperature was the dominant factor influencing response to acute high temperature for the previously noted processes.

**FIGURE 2:**
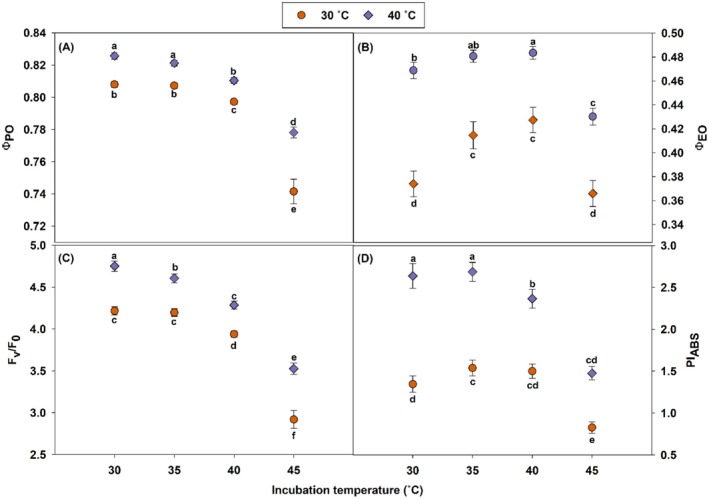
Effect of incubation temperature (30°C, 35°C, 40°C, and 45°C) and growth temperature (30°C/20°C and 40°C/30°C) on the maximum quantum yield of primary photochemistry (Φ_Po_; A), quantum yield of inter‐photosystem electron transfer (Φ_Eo_; B), performance index representing contribution of light reactions to primary photochemistry (F_v_/F_0_; C), and photosystem II performance index based on light absorption (PI_ABS_; D) for Upland and Pima cotton species. Data shown as scatter points represent means ± standard errors; SE (*n* = 20; 2 runs × 5 replications × 2 cotton species). Values not sharing a common letter significantly differ (*p* < 0.05).

Cotton plants grown at 30°C/20°C and 40°C/30°C had the highest values of Φ_Po_ at incubation temperatures of 30°C and 35°C (Figure 2A). As the incubation temperature increased above 35°C, Φ_Po_ declined significantly in both growth temperature regimes. Relative to the 30°C incubation temperature, Φ_Po_ at 40°C declined by 1.3% and 1.8% for cotton plants grown under the 30°C/20°C and 40°C/30°C temperature regimes, respectively. A higher reduction was observed as incubation temperature increased to 45°C (30°C/20°C = 8.2% and 40°C/30°C = 5.8%). High‐temperature‐grown plants exhibited a less pronounced decline in Φ_Po_ at 45°C than plants in the optimal temperature regime. Furthermore, at all incubation temperatures, cotton plants grown at the higher temperature regime (40°C/30°C) had higher Φ_Po_ values than plants grown under the 30°C/20°C regime (Figure 2A).

Φ_Eo_ peaked at 35°C and 40°C incubation temperatures for cotton plants grown under both the 30°C/20°C and 40°C/30°C temperature regimes (Figure 2B). Increases in Φ_Eo_ of 14% and 3% were observed for optimum and high temperature‐grown plants, respectively, as incubation temperatures increased from 30°C to 40°C. Furthermore, at an incubation temperature of 45°C, Φ_Eo_ declined by 7.6% and 4.6% for the 30°C/20°C and 40°C/30°C growth temperatures, respectively, relative to the average peak values. At all incubation temperatures, higher ΦEo values were recorded for plants grown at 40°C/30°C than at the 30°C/20°C temperature regime (Figure 2B).

The temperature response of F_v_/F_0_ (Figure 2C) was similar to that of Φ_Po_ (Figure 2A), with increases in incubation temperature corresponding to decreases in F_v_/F_0_. However, the percent decline in F_v_/F_0_ with increasing incubation temperatures was much greater than that of Φ_Po_. The highest F_v_/F_0_ value for plants grown at 40°C/30°C was observed at 30°C and declined at higher incubation temperatures. Meanwhile, F_v_/F_0_ for plants grown at 30°C/20°C was highest at the 30°C and 35°C incubation temperatures and declined by 7% and 31% at 40°C and 45°C, respectively (Figure 2C). F_v_/F_0_ for the 40°C/30°C growth temperature regime declined by 3%, 10%, and 26% at 35°C, 40°C, and 45°C incubation temperatures, respectively, relative to 30°C. Similar to other thylakoid responses above (Figure 2A,B), growth temperature significantly influenced F_v_/F_0_, and consistently higher values were noted for the 40°C/30°C plants compared to the 30°C/20°C plants.

PI_ABS_ was significantly influenced by the interaction between incubation temperature and growth temperature (Table 3). PI_ABS_ for plants grown at 40°C/30°C peaked at 30°C, remained constant as temperature increased to 35°C, and thereafter declined significantly at higher temperatures (10% at 40°C and 44% at 45°C). Meanwhile, for the 30°C/20°C cotton plants, PI_ABS_ increased by 14% as the incubation temperature increased from 30°C, peaked at 35°C, remained stable at 40°C, and declined by 46% relative to its average peak value at the highest incubation temperature (45°C) (Figure 2D). Furthermore, PI_ABS_ was consistently higher for high‐temperature‐grown plants at all incubation temperatures.

## 4 Discussion

The frequency and duration of extreme heat waves are expected to increase because of climate change (Jagadish et al. 2021). This is a potential limitation to cotton stand establishment and early‐season seedling vigor, especially in the southeastern US, where cotton production is concentrated. Numerous studies have detailed the detrimental effects of heat stress on early‐season vegetative growth, reproductive development, and photosynthetic efficiency of cotton plants, including cultivar sensitivities to heat stress (Kaur, Snider, Parkash, et al. 2023; Virk et al. 2021; Adegbenro et al. 2025; Parkash et al. 2024; Reddy, Reddy, and Hodges 1992; Reddy et al. 2017; Reddy, Hodges, and Reddy 1992; Reddy et al. 1991). However, a knowledge gap exists in differentiating chronic and acute heat stress responses of specific thylakoid processes for the two most cultivated cotton species. To this end, our study evaluated the rapid and long‐term responses of high temperatures on OJIP‐derived chlorophyll fluorescence parameters in Upland and Pima cotton. By assessing both steady‐state responses to elevated growth temperature and transient responses to rapid thermal stress, we identified key thylakoid processes associated with potential interspecific differences in heat acclimation and stress sensitivity for Upland and Pima cotton (DP 1646 and DP 341, respectively). Because each species was represented by a single commercial cultivar (Hutmacher 2021; Hand et al. 2023; USDA‐AMS 2020), we interpret the species comparisons below at the cultivar level; this is considered further in the limitations.

The first hypothesis of this study was that cotton species would influence thylakoid responses of cotton seedlings under long‐term elevated growth temperatures. Our results support this claim, as some structural and functional OJIP parameters showed significant main and interaction effects of cotton species and growth temperature. In most cases where treatment effects were significant, cotton seedlings grown under the elevated‐temperature regime had higher values for structural indicators, quantum efficiencies, and performance indices. Where the temperature‐by‐genotype interaction was significant, under chronic heat stress, the Upland cotton (DP 1646) consistently exhibited higher acclimation potential than Pima (DP 341) for most parameters except Φ_Eo_. This is evidenced by higher DP 1646 values than DP 341, particularly under high‐temperature conditions. Growth temperature and cotton species did not affect relative antenna size (ABS/RC) or most reaction‐center specific fluxes (RC/CS_0_, ABS/RC, TR_0_/RC, and DI_0_/RC). Similar results were reported by Virk et al. (2021), where no heat stress effects were seen for RC/CS_0_, ABS/RC, and TR_0_/RC, suggesting that these processes are relatively insensitive to chronic heat stress in cotton seedlings.

In the current study, long‐term exposure to a 40°C/30°C day/night temperature regime increased PSI‐related parameters. For example, the indicator of the size of the pool of final electron acceptors of PSI (Δ_VIP_) and the electron flux per reaction center to PSI end acceptors (RE_o_/RC) both increased for Upland and Pima cotton (DP 1646 and DP 341, respectively). Increases in these parameters are generally interpreted as indicators of enhanced PSI acceptor‐side capacity, suggesting that heat stress increased PSI capacity to draw electrons more effectively from the intersystem chain (Strasser et al. 2004; Oukarroum et al. 2009; Ceppi et al. 2012). DP 1646 showed a greater increase than DP 341 for both parameters (Δ_VIP_: 44.4% in DP 1646 vs. 38.5% in DP 341; RE_o_/RC: 55.9% vs. 39.7%), indicating that the Upland cotton cultivar had higher PSI capacity under heat stress. Virk et al. (2021) indicated that Δ_VIP_ increased with increasing growth temperature, with the highest values reported at 35°C and 40°C. The rise in Δ_VIP_ and RE_o_/RC with heat stress likely reflects a compensatory enhancement of PSI electron transport, helping to balance electron flow under stress (Zhang and Sharkey 2009; Schrader et al. 2004; Havaux et al. 1991), and Upland cotton appeared more efficient than Pima in maintaining stable electron transport. In this context, “balancing electron flow” refers to maintaining redox equilibrium between the electron input from PSII and PSI's capacity to accept and process electrons. A sufficiently strong PSI electron sink may prevent upstream over‐reduction and alleviate electron pressure in the intersystem chain (Zhang and Sharkey 2009), and may also help limit the formation of reactive oxygen species, thereby complementing the antioxidant‐based responses that contribute to thermotolerance in cotton (Khan et al. 2026; Luqman et al. 2025). Foundational studies support this interpretation: Havaux et al. (1991) demonstrated that PSI reaction centers close when their acceptor side becomes saturated, even as PSII centers remain comparatively open, and Schrader et al. (2004) further showed that PSI plays a dominant role in regulating thylakoid redox state under thermal stress. Thus, the increases in Δ_VIP_ and RE₀/RC observed here likely represent compensatory adjustments that expand PSI's capacity to maintain electron flow under elevated temperatures. Upland cotton exhibited a greater increase in both Δ_VIP_ and RE₀/RC than Pima cotton, suggesting that Upland cotton more effectively expanded PSI electron‐sink capacity when exposed to elevated growth temperature, representing a key interspecific difference in heat‐acclimation potential. Although the effect of long‐term heat stress on Δ_VIP_ has not been widely reported, studies on other abiotic stresses have shown that it is a highly sensitive indicator of PSI activity (Ceppi et al. 2012; Oukarroum et al. 2009). Similarly, the increases in the quantum yield of PSI end electron acceptor reduction (Φ_Ro_) under high‐temperature conditions followed a trend similar to that of Δ_VIP_ and RE_o_/RC, supporting improved electron transport from PSI to the final electron acceptors in thylakoid reactions under elevated temperatures.

In photosynthetic tissues, when excess excitation energy resulting from stress cannot be used for photochemistry, plants often increase non‐photochemical quenching or rely on alternative mechanisms to prevent the accumulation of reactive oxygen species (Ledford and Niyogi 2005; Demmig‐Adams and Adams Iii 2006). These alternative mechanisms could include structural and functional adjustments in the photosystems, such as changes in antenna configuration or PSI:PSII stoichiometry (Allen 2002; Allen and Pfannschmidt 2000; Melis 1999), thereby redistributing excitation energy between photosystems and modifying the redox balance of electron transport. Non‐photochemical dissipation, quantified here as DI₀/RC, represents one major protective mechanism that plants commonly employ under abiotic stress. Although in our study, Φ_Po_ and DI₀/RC remained stable across growth temperatures for both species. The lack of change in DI₀/RC suggests that cotton seedlings did not depend on enhanced dissipation as a protective response to chronic heat. Instead, increases in the electron flux per RC to electron acceptors beyond Q_A_ (ET₀/RC) under heat stress further confirm that PSII reaction centers continued to efficiently drive electron transport beyond Q_A_, supporting preserved linear electron flow and strong coordination with downstream acceptors such as PSI. Kaur, Snider, Parkash, et al. (2023) showed that the maximum quantum yield of PSII (reported as F_v_/F_m_) was stable under increased growth temperature. Similarly, other studies have reported the stable nature of PSII under high temperatures (Law and Crafts‐Brandner 1999; Snider, Oosterhuis, and Kawakami 2010; Adegbenro et al. 2025; Snider et al. 2013). The results of our study are consistent with findings in other heat‐stressed plants, where both PSII and PSI have been considered heat‐tolerant (Virk et al. 2021; Sharkey 2005).

Additionally, PSII and intersystem electron transport revealed contrasting responses of Upland and Pima cotton to optimal and elevated temperatures. At the optimum temperature (30°C/20°C), Pima cotton exhibited a higher quantum yield of inter‐photosystem electron transfer (Φ_Eo_ = 0.47) than Upland (Φ_Eo_ = 0.44), indicating more efficient electron transport beyond Q_A_ under normal conditions. Under moderate heat (40°C/30°C), Upland increased Φ_Eo_ by 11%, reaching levels comparable to Pima, suggesting enhanced electron transport efficiency under elevated temperature. Also, the performance index representing the contribution of light reactions to primary photochemistry (F_v_/F_0_) remained stable in Upland, indicating that PSII donor‐side activity was maintained (Strasser et al. 2004; Strasser et al. 2000). In contrast, Pima showed a slight decline, reflecting mild donor‐side stress. This suggests that Upland's increased Φ_Eo_ under heat was probably supported by intact donor‐side function, while in Pima, the slight donor‐side limitation may have constrained improvements in electron transport beyond Q_A_.

Furthermore, a comparison of photosystem responses indicated that PSII maintained functional stability, whereas PSI performance improved significantly under chronic heat, especially in Upland cotton. For example, the performance index reflecting PSII function (PI_ABS_) did not change with growth temperature (Table 2), consistent with the stable quantum efficiency of PSII (Φ_Po_) and F_v_/F_0_. In contrast, the total performance index (PI_TOTAL_), which includes PSI activity, increased significantly under chronic high temperatures (by 130% in Upland and 79% in Pima). This indicates that more electrons were directed downstream toward PSI under heat stress. In other words, while PSII photochemistry remained stable under chronic heat stress, electron flow toward PSI was stimulated, leading to an overall increase in PI_TOTAL_, which was more evident in Upland cotton. These changes, especially when supported by similar results for parameters like Δ_VIP_, RE_o_/RC, and Φ_Ro_ agree with earlier studies suggesting that moderate heat may stimulate electron transport beyond Q_A_ (Virk et al. 2021) and sometimes promote cyclic electron flow (Sharkey 2005; Zhang and Sharkey 2009), although the latter pathway was not directly assessed in our study. Also, to our knowledge, few studies have directly compared species‐level differences in thylakoid responses under chronic heat stress in cotton, particularly in terms of PSI activity. Our findings, therefore, provide evidence that Upland and Pima cotton may differ in their long‐term PSI performance. Prior cotton work using rapid induction fluorescence has mainly characterized cultivar‐ or genotype‐level variation, whether through field screening (Kaur, Snider, Paterson, et al. 2023), or controlled growth‐temperature regimes (Kaur, Snider, Parkash, et al. 2023; Adegbenro et al. 2025), typically using T_15_ thresholds for a limited set of parameters. Our study extends this to a between‐species comparison (Upland vs. Pima) across a broader set of OJIP parameters.

The second objective of this study was to assess the effects of growth temperature and cotton species on the sensitivity of specific thylakoid responses to acute high temperatures. Dark‐adapted OJIP measurements were obtained after exposure to rapid incubation temperatures to generate temperature response curves. For cotton plants grown under both optimal and elevated temperatures, the quantum yield of PSII (Φ_Po_) and some other indices (F_v_/F_0_, PI_ABS_) in response to acute heat stress were stable or peaked at moderate incubation temperatures (30°C–35°C), then declined at extreme temperatures (40°C–45°C). This pattern aligns with the known response of PSII to heat: moderate heat stress can temporarily enhance electron transport, whereas extreme temperatures lead to PSII inactivation (Hu et al. 2018; Kaur, Snider, Paterson, et al. 2023; Law and Crafts‐Brandner 1999). However, Φ_Eo_ peaked at slightly higher temperatures (35°C–40°C), suggesting that the intersystem electron transport chain was more tolerant to acute high temperature than the photochemical reactions of PSII. This appears to contrast with other studies where Φ_Eo_ was more heat sensitive compared to PSII (Snider et al. 2015; Adegbenro et al. 2025; Hu et al. 2018; Kaur, Snider, Paterson, et al. 2023). While we do not have a clear mechanistic explanation for these trends, there are some methodological differences between the current study and previous chlorophyll fluorescence‐temperature response experiments. In the current experiment, measurements were done in a leaf chamber with leaves remaining attached to the plant. However, most acute temperature response experiments are done with detached leaves or leaf segments placed directly on a moist, heated surface rather than exposing the leaf tissue to the surrounding air (Adegbenro et al. 2025; Kaur, Snider, Paterson, et al. 2023; Hu et al. 2018).

Furthermore, cotton plants grown at 40°C/30°C consistently showed higher values for these four parameters (Φ_Po_, Φ_Eo_, F_v_/F_0_, PI_ABS_) across all acute incubation temperatures, compared to plants grown under the 30°C/20°C regime. In addition to these higher absolute values, plants exposed to higher growth temperatures exhibited smaller percentage declines in these parameters as incubation temperature increased from 30°C to 45°C. For example, Φ_Po_ declined by 8.2% in 30°C/20°C plants but by only 5.8% in 40°C/30°C plants at 45°C, and similar patterns were observed for Φ_Eo_, F_v_/F_0_, and PI_ABS_. This implies that prior exposure to elevated growth temperatures enhanced the acute thermotolerance of these thylakoid responses (Berry and Bjorkman 1980). Possible explanations for greater thermotolerance under elevated growth temperatures include increased expression of heat shock proteins, stabilization of D1 protein repair dynamics, and membrane lipid remodeling (Chen et al. 2017; Heckathorn et al. 1998; Rokka et al. 2000; Theis and Schroda 2016; Higashi et al. 2018). This is well documented in the literature, where plants grown under warmer or cooler temperatures tend to show higher or lower thylakoid thermotolerance, respectively, compared to plants grown under normal conditions (Adegbenro et al. 2025; Kaur, Snider, Parkash, et al. 2023; Hu et al. 2018; Snider et al. 2013). The sustained PSII efficiency under acute heat stress in warm‐grown plants follows a pattern similar to that observed with chronic heat acclimation in objective one, in which Φ_Po_ remained stable, and DI₀/RC was unaffected despite long‐term thermal stress, suggesting preserved photochemical integrity. Field data reported by Snider et al. (2013) support the notion that PSII can acclimate to prevailing air temperature conditions. Leaves exposed to more prolonged and intense high‐temperature environments consistently exhibited higher T_15_ values (temperatures causing a 15% decline in thylakoid response), indicating improved PSII thermal tolerance. This increase in thermotolerance was positively correlated with ambient maximum temperatures, reinforcing the role of prior heat exposure in enhancing PSII resilience. Furthermore, controlled‐environment studies showed that increasing growth temperatures from 20°C/15°C to 40°C/30°C progressively raised the T_15_ thresholds of Φ_Po_ and Φ_Eo_, with each 1°C increase in maximum daily temperature resulting in approximately 0.33°C and 0.39°C increases in T_15_Φ_Po_ and T_15_Φ_Eo_, respectively (Adegbenro et al. 2025). Similarly, Kaur, Snider, Parkash, et al. (2023) reported that cotton plants grown at early‐season growth temperatures of 40°C/30°C exhibited higher temperature thresholds for Φ_Po_ than plants grown at 30°C/20°C.

Irrespective of growth temperature, PSI‐related parameters (Φ_Ro_ and PI_TOTAL_) increased from 30°C up to an optimum (35°C–40°C) and then collapsed at 45°C (Figure 1A,B). In contrast to the PSII‐centered parameters, Φ_Ro_ and PI_TOTAL_, which are more closely associated with PSI acceptor‐side activity, showed a delayed peak (around 40°C) before collapsing at 45°C, specifically for Upland cotton. This suggests that PSI‐end electron sinks and downstream acceptor capacity might be more thermally stable than PSII, which is consistent with the widely reported increases in PSI activity under moderate heat stress (Sun et al. 2018; Schrader et al. 2004; Boucher et al. 1990; Havaux et al. 1991). However, results from Kaur, Snider, Paterson, et al. (2023) and Hu et al. (2018) showed that both PSI and PSII did not differ significantly in thermotolerance. These contrasting outcomes likely reflect differences in experimental design. In our study, acute temperature responses were obtained from leaves that remained attached to intact plants and were heated by increasing the growth chamber air temperature. Although the exposure was acute, heating occurred via warm air rather than direct contact with a hot surface, allowing normal water and metabolic exchange during the temperature rise. In contrast, Hu et al. (2018) and Kaur, Snider, Paterson, et al. (2023) used leaf discs placed directly on a heated surface, resulting in much faster and more uniform tissue heating. Such rapid heating in those studies could have accelerated PSII inactivation and reduced the likelihood of detecting the enhanced PSI stability observed under more physiologically representative heating conditions, as in our study.

Upland cotton, which maintained higher Φ_Ro_ and PI_TOTAL_ at 40°C compared to Pima (35°C), had a higher thermal optimum for PSI‐related processes. This higher optimum is consistent with our chronic heat results, in which Upland showed a greater enhancement of PSI acceptor‐side capacity (e.g., increases in Δ_VIP_, RE₀/RC, and Φ_Ro_) under long‐term elevated temperatures compared with Pima. The acute temperature responses reinforce our findings on chronic heat, as the same PSI‐related adjustments (specifically, greater PSI electron‐sink capacity and enhanced downstream electron transport) are evident under both long‐term acclimation and short‐term thermal stress. Upland's higher thermal optimum for Φ_Ro_ and PI_TOTAL_ follows the observed increases in Δ_VIP_, RE₀/RC, and Φ_Ro_, suggesting that the acclimation established under sustained heat directly contributes to improved PSI function when plants encounter sudden high temperatures. This agreement across time demonstrates that Upland's PSI machinery is more flexible and maintains electron‐sink capacity more effectively under heat, supporting a species‐level difference in thermal resilience (Adegbenro et al. 2025). Our results contrast with studies reporting greater PSI thermotolerance in Pima (Kaur, Snider, Paterson, et al. 2023; Kaur, Snider, Parkash, et al. 2023). Notably, Kaur, Snider, Parkash, et al. (2023) found that Pima exhibited the lowest tolerance for growth and PSII‐related processes under high temperature, suggesting that PSI‐based thermotolerance alone did not translate to greater whole‐plant heat tolerance. These mixed patterns may reflect methodological differences: prior studies used detached leaf discs heated rapidly and uniformly on a hot surface, whereas our study assessed intact leaves on whole plants. As discussed previously, such rapid, uniform heating can alter PSI‐PSII comparative responses relative to the more gradual, physiologically representative heating of intact leaves. Taken together, our findings indicate that Upland cotton is more thermotolerant and is better equipped to maintain photosynthetic performance under both acute and prolonged heat stress.

A limitation of this study is that each species was represented by a single cultivar (DP 1646 B2XF and DP 341 RF). As a result, species‐level and cultivar‐level effects cannot be fully disentangled within the present design, and the differences reported here may partly reflect cultivar‐specific variation. Although the selected cultivars are commercially grown in the United States (Hutmacher 2021; USDA‐AMS 2020; Hand et al. 2023), confirming species‐level generalizations would require evaluation of multiple cultivars per species (Iqbal et al. 2019). The present findings should therefore be interpreted as cultivar‐level evidence consistent with, rather than definitive proof of, species‐level differences in thermotolerance, providing a basis for future multi‐cultivar studies.

## 5 Conclusions

Our findings demonstrate that acute heat stress responses closely mirrored patterns observed under chronic heat exposure, indicating that similar thylakoid adjustments operate across timescales in cotton. PSII photochemical efficiency was largely maintained under both conditions in Upland and Pima (DP 1646 and DP 341, respectively). In contrast, PSI‐related parameters were highly temperature‐responsive and were much enhanced under heat, particularly in DP 1646. DP 1646 exhibited more substantial increases in PSI acceptor‐side capacity (e.g., Δ_VIP_, RE₀/RC, Φ_Ro_) and higher thermal optima for Φ_Ro_ and PI_TOTAL_, alongside sustained electron transport beyond the primary plastoquinone acceptor (Q_A_). These coordinated responses suggest that DP 1646 is more flexible in maintaining donor‐side function, redirecting electron flow, and supporting PSI under heat stress, reflecting a more robust long‐term acclimation strategy, while DP 341 exhibited weaker PSI adjustments. Together, these results provide new insight into how cotton partitions thermal tolerance between the two photosystems and highlight the importance of PSI‐related acclimation in cotton. Because each species was represented by a single cultivar, studies incorporating multiple cultivars per species will be needed to determine whether the patterns observed here generalize to Upland and Pima cotton more broadly.

## Funding

This work was supported by Georgia Cotton Commission, Cotton Incorporated (23‐899), USDA National Institute of Food and Agriculture through the Hatch Research Capacity Fund, and USDA‐AFRI (2024‐67013‐42810).

## Conflicts of Interest

The authors declare no conflicts of interest.

## Supporting information


**Data S1:** OJIP chlorophyll *a* fluorescence dataset for Upland and Pima cotton seedlings grown under two day/night growth‐temperature regimes (30°C/20°C and 40°C/30°C).

**Data S2:** Rapid temperature‐response OJIP measurements on Upland and Pima cotton seedlings, grown at 30°C/20°C and 40°C/30°C were incubated at 30°C, 35°C, 40°C, and 45°C and re‐measured.

**File S1:** Documentation defining all data files, variables, abbreviations, units, and the software used.

**Table S1:** ANOVA summary of the effect of growth temperature (GT) and cotton species (CS) on chlorophyll fluorescence parameters (structural indicators, quantum efficiencies and performance indices).

## Data Availability

The data supporting the findings of this study are provided as Supporting Information with this article. This includes the raw chlorophyll *a* fluorescence datasets for the baseline (Data S1) and temperature‐response (Data S2) measurements, together with a README file describing all variables, units, and software (See File S1).
