# Supplementary material for: Investigating Thermotolerance of Thylakoid Processes in Two Cotton Species using Rapid Induction Fluorescence
Source: Plant Environ Interact. 2026 Jun 25;7(3):e70181. doi: 10.1002/pei3.70181 (PMC13296587; doi:10.1002/pei3.70181)
Supplement: Supplementary file 3 — File S1: Documentation defining all data files, variables, abbreviations, units, and the software used. [file PEI3-7-e70181-s004.docx]

**Supplementary File: README for Data from Investigating Thermotolerance of Thylakoid processes in two cotton species using rapid induction fluorescence**

**Description of the data and file structure**

These data were collected to characterize how chronic and acute high temperatures affect thylakoid-level photosynthetic processes in Upland (*Gossypium hirsutum*, cv. DP 1646 B2XF) and Pima (*G. barbadense*, cv. DP 341 RF) cotton. Four-week-old seedlings grown under two day/night temperature regimes (30/20 and 40/30 °C) were assessed using rapid induction chlorophyll *a* fluorescence (OJIP). Baseline measurements were taken under each growth temperature, followed by rapid temperature-response measurements (incubation at 30, 35, 40, and 45 °C) to evaluate the acclimation potential of photosystem II and photosystem I processes. The experiment was conducted across two experimental runs in a controlled environment.

**Files and variables**

**File: Supplementary Data S1.xlsx**

**Description:** Baseline rapid induction chlorophyll *a* fluorescence (OJIP) measurements for four-week-old Upland (DP 1646 B2XF) and Pima (DP 341 RF) cotton seedlings grown under two day/night growth-temperature regimes (30/20 and 40/30 °C), measured at the growth temperature across two experimental runs. Each row is a single leaf measurement; columns give sample identifiers, the raw fluorescence transient values (O, J, I, P), and derived OJIP-test parameters.

**Variables (all OJIP parameters are dimensionless ratios unless noted):**

- RUN- experimental run (1 or 2)
- Temperature- day/night growth-temperature regime, °C (30/20 or 40/30)
- Genotype- cotton genotype: DP 1646 B2XF (Upland, *Gossypium hirsutum*) or DP 341 RF (Pima, *G. barbadense*)
- Rep- replicate number within run
- Sample #- sample identifier
- O- minimal fluorescence (F₀) at the O step; relative fluorescence units
- J- fluorescence at the J step; relative fluorescence units
- I- fluorescence at the I step; relative fluorescence units
- P- maximal fluorescence (Fₘ) at the P step (peak); relative fluorescence units
- Mo- approximated initial slope of the transient (net rate of reaction-center closure)
- Fv/Fm- maximum quantum yield of primary PSII photochemistry
- Fv/Fo- performance index representing contribution of light reactions to primary photochemistry
- VJ- relative variable fluorescence at the J step
- VI- relative variable fluorescence at the I step
- ABS/RC- absorption flux per active reaction center
- TRo/RC- trapped energy flux per reaction center
- DIo/RC- dissipated energy flux per reaction center
- ETo/RC- electron transport flux beyond Q_A_ per reaction center
- REo/RC- electron flux reducing PSI end electron acceptors per reaction center
- Phi po- maximum quantum yield of primary photochemistry
- Psi Eo- probability a trapped exciton moves an electron beyond Q_A_
- Phi Eo- quantum yield of electron transport
- delta Ro- efficiency of electron transfer from intersystem carriers to PSI end acceptors
- Phi Ro- quantum yield of reduction of PSI end electron acceptors
- TRo/ABS- trapping probability
- ETo/TRo- electron transport probability
- ABS/CSo- absorption per excited cross-section at t=0
- ABS/CSm- absorption per excited cross-section at Fₘ
- TRo/CSo- trapped energy flux per cross-section
- ETo/CSo- electron transport flux per cross-section
- RC/CSo- density of active reaction centers per cross-section (t=0)
- RC/CSm- density of active reaction centers per cross-section (at Fₘ)
- PIabs- photosystem II performance index based on light absorption
- PItotal- total performance index representing energy conservation from the absorbed photons to a reduction of PSI end acceptors
- delta VIP (ΔVIP)- indicator of the size of pool of the final electron acceptors of PSI

**File: Supplementary Data S2.xlsx**

**Description:** Rapid temperature-response (acute) OJIP measurements, in which leaves were incubated at 30, 35, 40, and 45 °C and re-measured. Column structure matches the baseline file, with additional columns for incubation temperature and measured leaf temperature. Each row is a single leaf measurement at a given incubation temperature.

**Variables- identical to the baseline file, plus:**

- Temp_Gen- combined growth-temperature × genotype grouping code
- Incubation temperature- acute incubation temperature applied before measurement, °C (30, 35, 40, or 45)
- Tleaf- measured leaf temperature during measurement, °C

**Code/software**

The data files are provided in Microsoft Excel (.xlsx) format and can be opened with any spreadsheet software, including free options such as LibreOffice Calc or Google Sheets, or exported to CSV. No proprietary software is required to view the data. Statistical analyses were performed in JMP Pro 18 (SAS Institute), and graphs were produced in SigmaPlot 16.0. No analysis scripts are included in this submission
