# Supplementary material for: Investigating Thermotolerance of Thylakoid Processes in Two Cotton Species using Rapid Induction Fluorescence
Source: Plant Environ Interact. 2026 Jun 25;7(3):e70181. doi: 10.1002/pei3.70181 (PMC13296587; doi:10.1002/pei3.70181)
Supplement: Supplementary file 4 — Table S1: ANOVA summary of the effect of growth temperature (GT) and cotton species (CS) on chlorophyll fluorescence parameters (structural indicators, quantum efficiencies and performance indices). [file PEI3-7-e70181-s001.docx]

**Supplementary Table S1:** ANOVA summary of the effect of Effect of growth temperature (GT) and cotton species (CS) on the density of PSII reaction centers (RC/ CSo), absorbed energy flux per RC (ABS /RC), flux of excitation energy trapped by a reaction center (TRo/RC), electron flux per RC to electron acceptors beyond QA (ETo/RC), electron flux per RC to PSI end electron acceptors (REo/RC), dissipation energy flux per RC (DIo/RC), indicator of the size of pool of the final electron acceptors of PSI (Δ_VIP_), maximum quantum yield of primary photochemistry (Φ_Po_), quantum yield of inter-photosystem electron transfer (Φ_Eo_), and quantum yield of reduction of PSI end electron acceptors (Φ_Ro_), performance index representing contribution of light reactions to primary photochemistry (F_v_/F_0_), photosystem II performance index based on light absorption (PI_ABS_), and performance index representing energy conservation from the absorbed photons to a reduction of PSI end acceptors (PI_TOTAL_). Values represent p-values at a significance level of 0.05.

|  |  | **p-values** | | |
| --- | --- | --- | --- | --- |
| **Category** | **Parameter** | **GT** | **CS** | **GT × CS** |
| **Structural Indicators** | **RC/CS_o_** | 0.6784 | 0.6211 | 0.6489 |
|  | **ABS/RC** | 0.3677 | 0.7804 | 0.575 |
|  | **TR_o_/RC** | 0.4291 | 0.6791 | 0.4435 |
|  | **DI_o_/RC** | 0.2157 | 0.7792 | 0.7692 |
|  | **ET_o_/RC** | **0.021** | 0.4364 | 0.0991 |
|  | **RE_o_/RC** | **<0.0001** | **0.0135** | **0.0389** |
|  | **Δ_VIP_** | **<0.0001** | **<0.0001** | **0.0008** |
| **Quantum Efficiencies** | **Φ_Po_** | 0.8619 | 0.1778 | 0.7894 |
|  | **Φ_Eo_** | **0.0042** | **0.0127** | **0.0066** |
|  | **Φ_Ro_** | **<0.0001** | **<0.0001** | **0.0001** |
| **Performance Indices** | **F_v_/F_0_** | 0.1008 | 0.1448 | **0.0088** |
|  | **PI_ABS_** | 0.22 | 0.1119 | 0.106 |
|  | **PI_TOTAL_** | **<0.0001** | **<0.0001** | **<0.0001** |
